# 834. Fetal Bovine Dermal Scaffold in the Management of Full and Partial Thickness Burn Injuries

**DOI:** 10.1093/jbcr/irag033.273

**Published:** 2026-04-06

**Authors:** Victoria Young, Kristy Miller, Talia Selembo, Alfredo C Cordova

**Affiliations:** Sarasota Memorial Hospital, Sarasota, FL; Sarasota Memorial Hospital, Sarasota, FL; Sarasota Memorial Hospital, Sarasota, FL; Sarasota Memorial Hospital, Sarasota, FL

## Abstract

**Introduction:**

Managing partial and full thickness burn wounds are often complicated by extensive devitalized tissue, tissue loss, and heavy colonization or infection. While skin substitutes aid in temporary coverage and wound bed preparation, many are susceptible to bacterial colonization. Notably, fetal bovine dermal scaffold has shown to support healing in both partial and full thickness wounds of various etiologies, despite these challenges.

**Methods:**

Twenty-three patients with full and partial thickness wounds from burn injuries were treated with fetal bovine dermal scaffold, either alone or prior to autografting. Our protocol included surgical wound excision, bed preparation, and infection control prior to dermal scaffold application and use of negative pressure wound therapy. Most of our patients had significant comorbidities. Outcomes assessed included wound bed readiness, scaffold integration, and infection rates.

**Results:**

Mean TBSA for the partial thickness group was 6% while for the full thickness cohort was 9%. Complete xenograft incorporation and a vascularized wound bed optimal for autografting was attained within 14-21 days. All patients tolerated the scaffold without adverse reactions. No graft loss or infection was recorded. Autologous STSG-take was >95% with complete epithelization within 2-weeks.

**Conclusions:**

Fetal bovine dermal scaffold facilitated early neovascularization and granulation tissue formation. It is a safe and effective adjunct in managing complex wounds, particularly in patients with comorbidities or high infection risk. It enhances wound bed preparation, reduces time to grafting, and may improve overall outcomes. Larger cohort studies may be required to validate our experience.

**Applicability of Research to Practice:**

Fetal bovine dermal scaffold may facilitate and accelerate healing of both partial and full thickness burn wounds.

**Funding for the study:**

N/A.

